# MRDviz: an integrated platform for interactive visualization and joint simulation of minimal residual disease trajectories and associated survival outcomes

**DOI:** 10.1093/bioadv/vbag029

**Published:** 2026-01-30

**Authors:** Kwangbom Choi, Kevin Zhao, Tabrez A Mohammad, Paul M Jung, Shiquan Wu, Jifeng Qian, Paul Hiser, Edith Szafer Glusman, Leo Wang-Kit Cheung

**Affiliations:** Bioinformatics and Computational Oncology, Quantitative Medicine & Genomics, AbbVie Company, 1000 Gateway Blvd., South San Francisco, CA, 94080, United States; Bioinformatics and Computational Oncology, Quantitative Medicine & Genomics, AbbVie Company, 1000 Gateway Blvd., South San Francisco, CA, 94080, United States; Bioinformatics and Computational Oncology, Quantitative Medicine & Genomics, AbbVie Company, 1000 Gateway Blvd., South San Francisco, CA, 94080, United States; Bioinformatics and Computational Oncology, Quantitative Medicine & Genomics, AbbVie Company, 1000 Gateway Blvd., South San Francisco, CA, 94080, United States; Bioinformatics and Computational Oncology, Quantitative Medicine & Genomics, AbbVie Company, 1000 Gateway Blvd., South San Francisco, CA, 94080, United States; Bioinformatics Engineering, Quantitative Medicine & Genomics, AbbVie Company, 1000 Gateway Blvd, South San Francisco, CA, 94080, United States; Information Research, AbbVie Company, 1000 Gateway Blvd., South San Francisco, CA, 94080, United States; Precision Medicine, Oncology, AbbVie Company, 1000 Gateway Blvd., South San Francisco, CA, 94080, United States; Bioinformatics and Computational Oncology, Quantitative Medicine & Genomics, AbbVie Company, 1000 Gateway Blvd., South San Francisco, CA, 94080, United States

## Abstract

**Motivation:**

Minimal residual disease (MRD) assessment has become a powerful tool in modern cancer management, offering early, actionable insights across diverse malignancies and often anticipating clinical events traditionally used to gauge therapeutic response. Beyond quantifying residual disease burden, MRD monitoring enables earlier intervention, nuanced treatment decisions, and improved outcome prediction before conventional endpoints appear. MRD negativity is now recognized as a potential surrogate endpoint, informing regulatory frameworks and accelerating drug development, as reflected by the FDA’s approval of MRD as an accelerated endpoint in Multiple Myeloma trials. In particular, longitudinal MRD assessments, which track dynamic changes over time rather than single time points, have shown enhanced prognostic value, and more trials are now collecting such data.

**Results:**

Building on this progress, we present MRDviz, an integrated R Shiny platform for exploratory analysis and simulation of longitudinal MRD data alongside survival outcomes. MRDviz offers interactive visualizations of MRD trajectories, real-time data quality checks, and identification of patient subgroups with distinct outcomes. Through coordinated views, customizable filtering, and integrated survival analyses, MRDviz empowers researchers to refine clinical hypotheses, design robust studies, and enhance the interpretation of MRD data, ultimately supporting better clinical decision-making and patient care.

**Availability and implementation:**

MRDviz is freely available at https://github.com/abbvie-external/MRDviz.

## 1 Introduction

Minimal residual disease (MRD) refers to the small number of cancer cells that may remain in a patient after treatment, which are often undetectable by standard imaging techniques. The ability to detect these residual cells can provide critical insights into disease progression and therapeutic efficacy, facilitating risk stratification and enabling the optimization of treatment strategies (Schuurhuis *et al.* 2018, Jain *et al.* 2024, El Chaer *et al.* 2025, Short and Dillon 2025). Modern molecular techniques such as multicolor flow cytometry (Sun *et al.* 2025), next-generation sequencing (Ching *et al.* 2020), and circulating tumor DNA (ctDNA) analysis (Newman *et al.* 2014, Kurtz *et al.* 2021) have revolutionized MRD detection, allowing for remarkable sensitivity (Levin *et al.* 2018). These methods can identify a single cancer cell (or equivalent genomic copy of ctDNA) among millions of healthy cells, making them invaluable not only in hematologic malignancies like acute lymphoblastic leukemia (ALL) and multiple myeloma (MM) (Munshi *et al.* 2017, Saygin *et al.* 2022, Dekker *et al.* 2023), where MRD status strongly correlates with prognosis, but also in solid tumors such as colorectal (Tie *et al.* 2016, 2019, Parikh *et al.* 2021) and lung cancer (Pantel and Alix-Panabières 2025), where emerging MRD assays are demonstrating potential for guiding treatment decisions and predicting recurrence.

Longitudinal measurements of MRD provide even richer insights into temporal dynamics of the treatment effect and disease evolution compared to single time-point assessments. By capturing dynamic patterns over the course of treatment, MRD monitoring enables more accurate predictions of patient outcomes and timely therapeutic adjustments (Gu *et al.* 2018, Blombery and Cheah 2022, Garcia-Murillas *et al.* 2025). However, fully realizing the potential of longitudinal MRD data requires rigorous attention to quality assurance, visual representation, statistical modeling, and clinical applicability assessment. Inconsistent measurements or analytical artifacts can introduce uncertainty into MRD interpretations, potentially affecting its impact on clinical outcomes and decision-making. By enabling real-time visual checks, researchers and clinicians can more effectively identify and address data quality issues at an early stage and test hypotheses on the fly. Investigators can rapidly test the plausibility of candidate biomarkers, refine patient stratification, and improve trial design decisions. This proactive approach enhances the credibility and clinical utility of MRD-based insights, ensuring that they better inform therapeutic strategies and patient care.

Exploratory data analysis is essential (Gelman 2004, Irizarry and Love 2021) in biomarker research, where complex, multidimensional datasets can obscure subtle patterns and confounding factors. In the context of MRD, where longitudinal patterns may be shaped by diverse biological and clinical variables, interactive visualization tools are especially valuable, allowing researchers to dynamically investigate relationships and rapidly test different modeling strategies or data transformations. To address these demands, we developed MRDviz, an interactive visualization platform specifically designed for longitudinal MRD data exploration in combination with patients’ survival data. MRDviz prioritizes quality control and enables early detection of measurement inconsistencies, batch effects, and anomalous patterns, thereby improving the reliability of subsequent analyses and clinical interpretations. With its dynamic filtering capabilities, real-time association analyses, and flexible simulation components for generating synthetic datasets, MRDviz supports hypothesis generation as well as hypothesis testing, quality assurance, and methodological innovation. By facilitating a more nuanced understanding of MRD dynamics, MRDviz provides an adaptable foundation for advancing MRD research and ultimately enhancing patient care.

## 2 Methods

### 2.1 Integrated, interactive visualization of longitudinal and time-to-event data

**Figure 1:**
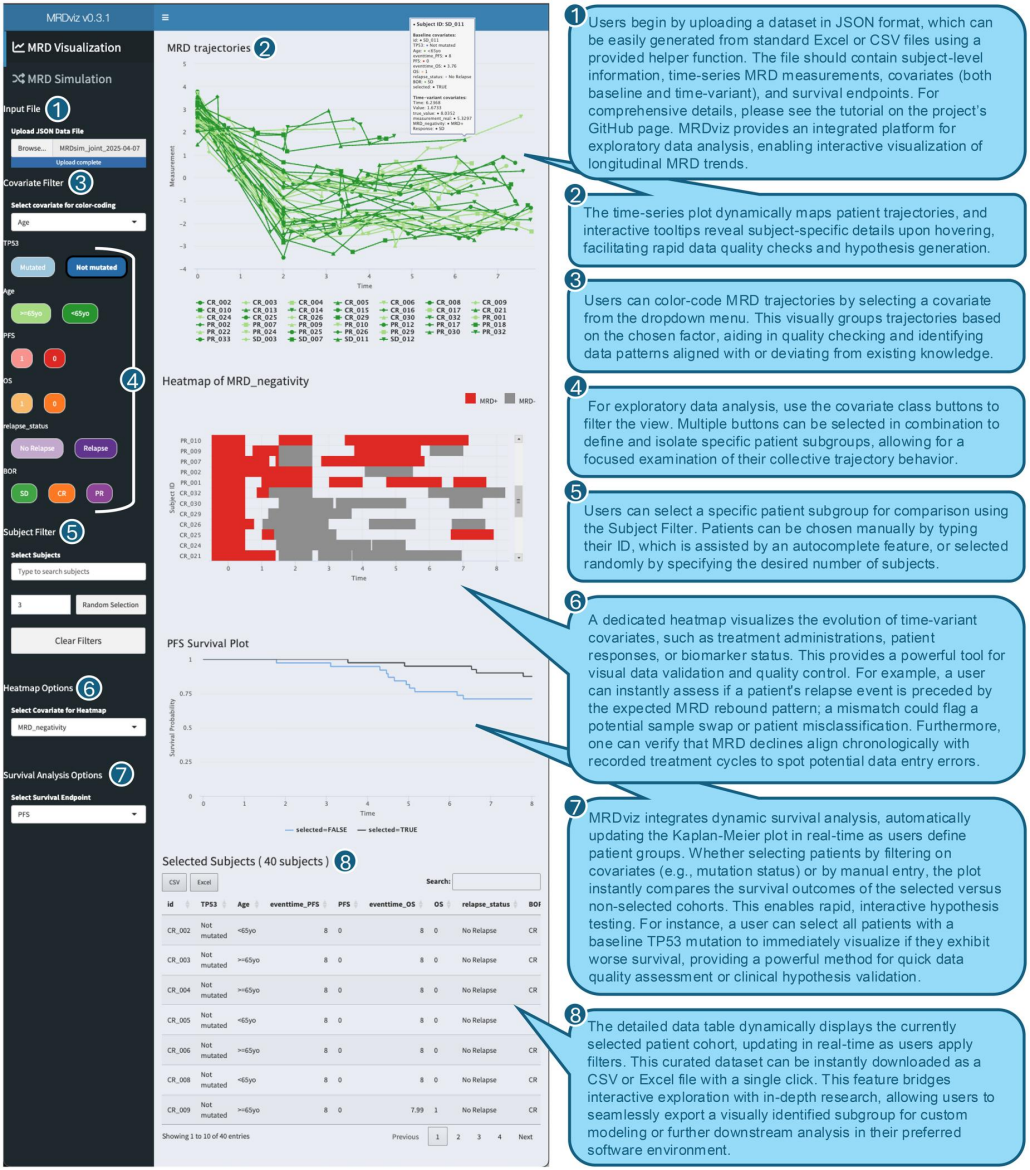
The MRDviz platform for interactive visualization and exploratory analysis. A screenshot of the MRDviz interactive visualization interface, designed for the exploratory data analysis of longitudinal Minimal Residual Disease (MRD) data alongside associated survival outcomes. The numbered callouts highlight the typical user workflow, which includes uploading data, applying filters to isolate patient subgroups, and using the coordinated plots to visually inspect trajectories, time-variant covariates, and survival patterns in real-time.

MRDviz is a specialized Shiny web application that provides researchers and clinicians with an interactive and intuitive environment (Figure 1) for analyzing complex longitudinal MRD data alongside survival outcomes. Users can upload a JavaScript Object Notation (JSON) file containing subject-level information, time-series MRD measurements, covariates (both baseline and time-variant), and survival endpoints, facilitating efficient data exploration through interactive visualizations. The user interface, built with shiny dashboard, features a structured layout with a sidebar for file uploading, covariate selection, subject filtering, and random sampling. Users can refine their analysis by selecting patient subgroups through dynamically generated tags representing baseline covariates, allowing rapid isolation of patient cohorts based on genetic markers, demographic attributes, or clinical features. Designed with accessibility in mind, MRDviz serves both experienced data scientists and clinicians with limited programming expertise. Color plays a crucial role in pattern recognition within MRDviz. The application integrates the RColorBrewer package to implement an advanced color management system that enhances interpretability. Binary covariates are assigned contrasting color pairs, while categorical variables with multiple levels utilize color ramps to create visually coherent sequences. This standardized approach helps users track patterns, identify outliers, and compare subgroups effectively. Additionally, users can specify custom hex-code colormaps in the JSON input file if needed.

MRDviz offers multiple visualization components to explore longitudinal MRD trends. The timeseries line plots dynamically map patient MRD trajectories to selected covariates. For example, users can filter male patients while color-coding their trajectories based on TP53 mutation status. Interactive tooltips provide additional context, revealing subject-specific baseline and time-variant details upon hovering. A dedicated heatmap visualization presents time-variant covariates such as treatments, patient responses, and biomarker status, offering a high-level view of how these factors evolve over time. All baseline and time-variant covariates are automatically processed and displayed in the sidebar, allowing users to seamlessly interact with the dataset.

Beyond MRD trajectory analysis, MRDviz integrates survival analytics, linking longitudinal data to clinical endpoints. The application processes all endpoint data automatically, enabling users to select from a drop-down menu. Kaplan-Meier curves are then generated to compare primary endpoint probabilities between selected and non-selected patient groups. This feature allows researchers to assess whether specific MRD patterns or covariate profiles are associated with different survival outcomes, providing deeper insights into disease progression and treatment effectiveness.

A dynamically filtered data table, implemented using the DT package, complements the graphical visualizations by offering direct access to the underlying dataset. This feature enhances transparency and supports reproducible research by allowing users to examine numerical values, identify specific subjects, and export filtered datasets for further statistical modeling or validation. By integrating data filtering, visualization, and inspection into a seamless workflow, MRDviz promotes iterative and exploratory data analysis.

By integrating robust data management, flexible visualization, and advanced statistical methods, MRDviz empowers users to uncover subtle MRD patterns, identify potentially prognostic covariates, and generate insights that inform patient stratification, treatment decisions, and future research. The result is a scientifically rigorous yet user-friendly tool that enhances the understanding of MRD trajectories and their implications for patient outcomes in hematological malignancies and solid tumors.

### 2.2 Simulation of MRD trajectories and an associated time-to-event data

**Figure 2:**
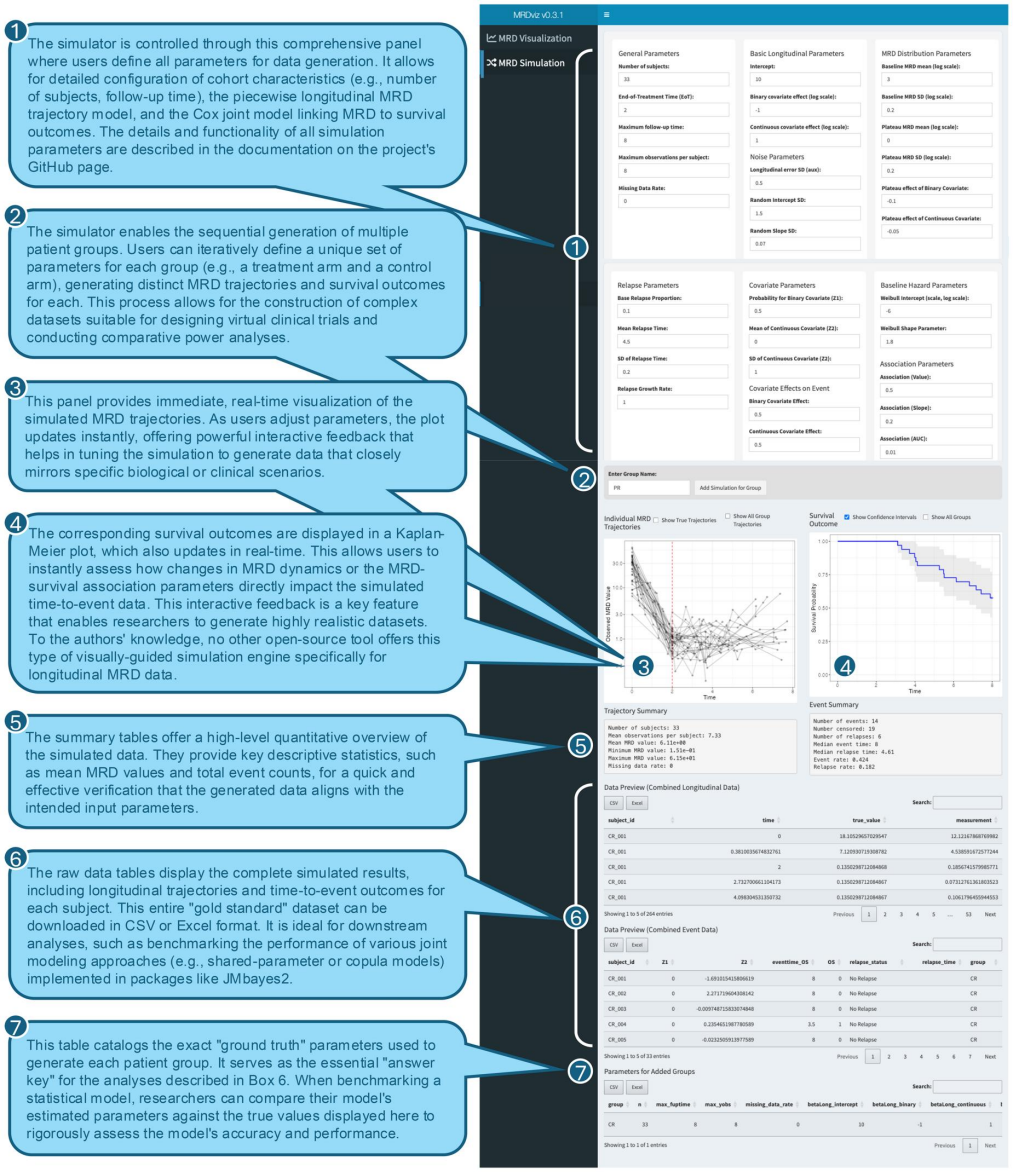
The MRDviz interface for joint simulation of MRD trajectories and survival outcomes. A screenshot of the MRDviz simulation module, which generates synthetic “gold standard” datasets by jointly modeling longitudinal MRD trajectories and time-to-event data. The numbered callouts illustrate the simulation workflow, from defining model parameters and patient groups to the instant visualization of generated trajectories and survival curves. The lower panels show the complete data output, including quantitative summaries, the raw downloadable dataset, and the “ground truth” parameters used for the simulation, which are essential for benchmarking statistical models.

MRDviz features a robust simulation module (Figure 2) that enables researchers to generate synthetic MRD trajectories and associated survival outcomes using a joint modeling approach (Brilleman 2019). By adjusting parameters related to baseline characteristics, treatment effects, the MRD-survival association, sampling rate, and noise levels, users can explore hypothetical scenarios, evaluate statistical methods, and conduct power and sensitivity analyses. Built with Shiny, MRDviz offers an intuitive graphical user interface (GUI) that allows for real-time parameter adjustments and immediate visualization of data patterns and statistical summaries. Interactive visualizations, including MRD trajectory plots, Kaplan-Meier curves, and summary statistics, enhance interpretability, helping researchers assess alignment with study expectations. These capabilities make MRDviz a valuable tool for both methodological research and clinical trial planning.

The simulator models longitudinal MRD trajectories using a biologically plausible piecewise function with three distinct phases. The observed MRD value for patient *i* at time *t*, denoted as $y_{i}(t)$, is the sum of the true underlying trajectory, $\mu_{i}(t)$, and a normally distributed measurement error term, $\epsilon_{i}(t)∼N(0,\sigma^{2})$. The true trajectory $\mu_{i}(t)$ is defined by the following function:


$$\mu_{i}(t)=\{\begin{array}{c} \beta_{0i}\cdot \;\text{exp}\;\left(-\frac{\ln(\beta_{0i}/\beta_{1i})}{t_{e}}\cdot t\right)\;\text{if}\;t<t_{e}\\ \beta_{1i}\;\;\;\;\;\;\;\;\;\;\;\;\;\;\;\;\;\;\;\;\;\;\;\;\;\;\;\;\;\;\;\;\;\;\;\;\;\;\;\;\;\;\;\;\;\;\;\;\;\;\;\;\;\;\;\;\;\;\;\;\;\;\;\;\text{if}\;t_{e}\leq t\leq t_{r}\\ \beta_{1i}\cdot \;\text{exp}(\beta_{2}(t-t_{r}))\;\;\;\;\;\;\;\;\;\;\;\;\;\;\;\;\;\;\;\;\;\;\;\text{if}\;t>t_{r} \end{array}$$

Here, $\beta_{0i}$ is the patient-specific baseline MRD value, $\beta_{1i}$ is the plateau MRD level achieved at the end-of-treatment time ($t_{e}$), and $\beta_{2}$ is the exponential growth rate following relapse at time $t_{r}$. In the initial decay phase, MRD levels decline exponentially from baseline to end-of-treatment (EoT), with the rate of decline individualized for each patient. Following treatment completion, MRD stabilizes at a lower level (often below the limit of detection) in the plateau phase. In patients experiencing disease recurrence, MRD levels rise exponentially from the plateau level in the relapse phase. Each patient’s trajectory is drawn from distributions that reflect typical MRD dynamics: baseline MRD and plateau values follow lognormal distributions, while relapse times, when applicable, are sampled from normal distributions centered around a specified mean time point. By leveraging this piecewise framework, researchers can generate realistic MRD trajectories for subgroups defined by, for example, clinical response categories, such as Complete Responders (CR), Partial Responders (PR), Stable Disease (SD), and Progressive Disease (PD), with each subgroup assigned distinct baseline MRD distributions, EoT parameters, and cohort sizes. The tool also allows customization of MRD assessment schedules to simulate both densely and sparsely sampled datasets while accounting for biological variability and measurement noise.

This framework integrates seamlessly with survival modeling through Cox proportional hazards models. The hazard of an event for patient *i* at time *t*, $h_{i}(t)$, incorporates associations based on MRD level, rate of change, and cumulative disease burden, as defined by the equation:


$$\frac{h_{i}(t)}{h_{0}(t)}=\text{exp}\;\left(\gamma^{T}x_{i}+\alpha_{1}\mu_{i}(t)+\alpha_{2}\frac{d\mu_{i}(t)}{dt}+\alpha_{3}\int_{0}^{t}\mu_{i}(s)ds\right)$$

In this model, $h_{0}(t)$ is the baseline hazard function, $x_{i}$ is a vector of baseline covariates with corresponding log-hazard ratios γ, and the association parameters $\alpha_{1},\alpha_{2},$ and $\alpha_{3}$ quantify the risk associated with the current MRD level, its instantaneous rate of change, and the cumulative disease burden, respectively. This structure allows for realistic simulations across diverse patient populations and treatment scenarios.

The simulation capabilities of MRDviz make it highly applicable to clinical research and trial design. Sensitivity analyses can be performed by systematically varying key parameters, such as missing data patterns, the strength of MRD-survival associations, outlier presence, or subgroup characteristics, to assess the robustness of statistical models and trial conclusions. In addition, the simulator enables rigorous power analysis by estimating sample sizes and event rates required to detect treatment effects on survival. Unlike traditional analytical methods, this simulation-based approach accounts for complex, patient-specific variability, ensuring more reliable and trial-specific power calculations. MRDviz also helps optimize trial protocols by identifying the most effective measurement timing and frequency for MRD assessments, ensuring efficient data collection while maintaining statistical power. The joint simulator enables researchers to investigate MRD-survival association structures that may support MRD as a surrogate endpoint for long-term survival. Additionally, MRDviz facilitates the evaluation of the limit of detection (LoD) when defining MRD negativity in ctDNA studies, ensuring biomarker thresholds are appropriately set for accurate disease monitoring.

In conclusion, MRDviz provides a scientifically rigorous and flexible environment for simulating MRD trajectories and survival data. By integrating a biologically motivated piecewise model with interactive visualization and clinical trial modeling, it enables realistic dataset generation for hypothesis testing and clinical trial planning. Its ability to systematically vary key parameters supports sensitivity analyses, power calculations, and surrogacy evaluation, making it a valuable resource for refining statistical methods and optimizing study designs in MRD-driven research.

## 3 Concluding remarks

MRDviz is a valuable first-step tool for exploring structure in longitudinal biomarker data in conjunction with time-to-event outcomes. Its visualization and simulation modules promote intuitive exploration of MRD trajectories and associated covariates, enabling researchers and clinicians to more easily identify measurement inconsistencies, recognize interesting patient subgroups, and generate data-driven hypotheses for further investigation. Its flexible architecture, accommodating any longitudinal biomarker data linked to time-to-event outcomes, ensures broad applicability beyond MRD studies in areas like solid tumor or biomarker discovery. To our knowledge, no other tool provides (i) a platform for simultaneous exploratory data analysis of both MRD trajectories and survival outcomes, or (ii) an interactive simulation engine that allows users to generate realistic data to benchmark and compare different modeling strategies. MRDviz promotes a “human-in-the-loop” approach, fostering productive communication and bridging the gap between technical teams and clinical experts to enhance decision-making.

## Data Availability

The MRDviz R package is open-source and freely available on GitHub at https://github.com/abbvie-external/MRDviz under the MIT license. A permanent, archived version of the software (v0.3.1) corresponding to this manuscript is available on Zenodo at https://doi.org/10.5281/zenodo.16915302.
